# An empirical fuzzy multifactor dimensionality reduction method for detecting gene-gene interactions

**DOI:** 10.1186/s12864-017-3496-x

**Published:** 2017-03-14

**Authors:** Sangseob Leem, Taesung Park

**Affiliations:** Department of Statistics, Seoul National University, Seoul, 08826 South Korea

**Keywords:** Gene-gene interaction, Fuzzy set theory, Fuzzy MDR, Multifactor dimensionality reduction

## Abstract

**Background:**

Detection of gene-gene interaction (GGI) is a key challenge towards solving the problem of missing heritability in genetics. The multifactor dimensionality reduction (MDR) method has been widely studied for detecting GGIs. MDR reduces the dimensionality of multi-factor by means of binary classification into high-risk (H) or low-risk (L) groups. Unfortunately, this simple binary classification does not reflect the uncertainty of H/L classification. Thus, we proposed Fuzzy MDR to overcome limitations of binary classification by introducing the degree of membership of two fuzzy sets H/L. While Fuzzy MDR demonstrated higher power than that of MDR, its performance is highly dependent on the several tuning parameters. In real applications, it is not easy to choose appropriate tuning parameter values.

**Result:**

In this work, we propose an empirical fuzzy MDR (EF-MDR) which does not require specifying tuning parameters values. Here, we propose an empirical approach to estimating the membership degree that can be directly estimated from the data. In EF-MDR, the membership degree is estimated by the maximum likelihood estimator of the proportion of cases(controls) in each genotype combination. We also show that the balanced accuracy measure derived from this new membership function is a linear function of the standard chi-square statistics. This relationship allows us to perform the standard significance test using *p*-values in the MDR framework without permutation. Through two simulation studies, the power of the proposed EF-MDR is shown to be higher than those of MDR and Fuzzy MDR. We illustrate the proposed EF-MDR by analyzing Crohn’s disease (CD) and bipolar disorder (BD) in the Wellcome Trust Case Control Consortium (WTCCC) dataset.

**Conclusion:**

We propose an empirical Fuzzy MDR for detecting GGI using the maximum likelihood of the proportion of cases(controls) as the membership degree of the genotype combination. The program written in R for EF-MDR is available at http://statgen.snu.ac.kr/software/EF-MDR.

## Background

Investigating gene-gene and gene-environment interaction can be useful to understand genetic architecture of complex traits because most complex phenotypes are altered by multiple genes [1]. While many genome-wide association studies (GWAS) have successfully detected single nucleotide polymorphisms (SNPs) associated with phenotypes, focusing only on marginal effects of individual SNPs in complex traits could result in low power and replication rate in genetic association studies [2, 3]. Furthermore, the individual SNPs are not sufficient for explaining the global heritability of complex traits. This missing heritability may be caused by gene-gene interaction (GGI) or rare variants [4].

In genetic association studies, there are many different methods to analyze GGIs, such as regression modeling [5–8], pattern recognition [9, 10], data reduction [11–14], random forest [15] and support vector machine [16].

In an analysis of GGI for complex traits, one of the hurdle points lies in high dimensionality and difficulty in interpretation of interaction mechanism. For example, assume that the number of SNPs of interest is two. Then, there are 3 × 3 possible genotypes (cells) for biallelic SNPs. For a binary phenotype, there are 2^3×3^ possible interaction models including redundant models [17].

Among the GGI methods, the multifactor dimensionality reduction (MDR) method is known to be advantageous to identify high-order interactions [12, 18–20] and has been widely applied to detect GGIs in many common complex diseases (http://epistasis.org). The MDR method was developed for balanced case/control studies. MDR pools multiple genotypes into high-risk (H) and low-risk (L) groups depending on whether or not the number of cases is larger than the number of controls in a given genotype (a cell in a contingency table). MDR reduces a dimension of the genotypes into two H/L groups. Since MDR is a non-parametric approach, the best SNPs combinations are selected by the accuracy and consistency in a cross-validation procedure. For the detection of GGI in unbalanced datasets, Velez et al. [21] proposed a balanced accuracy function, and it has been widely used in MDR extensions.

Since its first introduction, many extensions of MDR have been proposed. The generalized MDR [22] was proposed by using a generalized linear model to overcome two drawbacks: the first is that MDR cannot adjust for covariates and the second is that MDR only can handle dichotomous traits. The pedigree-based GMDR [23] was proposed for the analysis of pedigree datasets using a transformation from a family data to a matched data. Later, the computing efficient version of the pedigree-based GMDR was proposed by Chen et al. [24] using the score-based statistic. Chen et al. [25] proposed the unified GMDR for the analysis of both family and unrelated data. GMDR was recently extended for the skewed data [26]. For the analysis of survival data, Beretta et al. [27] proposed the survival dimensionality reduction using a normalized mean time, Lee et al. proposed Cox-MDR [28, 29] using Cox-hazard model and Gui et al. [30] proposed Surv-MDR using a log-rank test.

Since MDR methods search causative SNP combinations in an exhaustive search manner, computation time increases exponentially by increases of a number of SNPs and an order of interactions. In order to reduce the execution time and computational burden, filtering methods such as Relief [31], TuRF [32] and SURF [33] can be adapted on a preprocessing step. Greene et al. [34] reduced the execution time using a graphic processing unit and Kwon et al. [35] improved computation time using the compute unified device architecture.

Despite its popularity, one of the shortcomings of MDR lies in its uncertainty of simple binary high(H)/low(L) classification. In MDR analysis, the binary classification compares the conditional odds of case and control given a genotype combination to the unconditional odds of the total numbers of cases and controls. Although this binary classification provides a straightforward interpretation of result, it suffers from a loss of information. Many extensions of MDR have concerned about this H/L binary classification of original MDR. For example, Model-based MDR [36] pools empty cells or the cells with similar numbers of cases and controls into a third risk-group ‘no evidence’ using chi-square tests. This method is extended to a unified modelling framework using the Wald test [37]. Robust MDR [38] uses a similar ‘unknown risk’ group using a Fisher’s exact test. Chung et al. proposed OR-MDR [39] using estimated odds ratios (ORs) as values of a quantitative trait risk for each genotype and Namkung et al. [40] proposed a weighting approach using OR of each genotype for computing the weighted balanced accuracy (wBA) in order to take into account of these differences [40].

Recently, we proposed a novel MDR extension, Fuzzy MDR [41], by using the fuzzy set theory. In Fuzzy MDR, we regard classifying high-risk group or low-risk group as equivalent to defining the degree of membership of two risk groups H/L. By adopting the fuzzy set theory, we proposed Fuzzy MDR which takes into account the uncertainty of H/L classification. Fuzzy MDR allows the possibility of partial membership of H/L through a membership function, which transforms the degree of uncertainty into a [0,1] scale. The best genotype combinations can be selected which maximizes a new fuzzy set based accuracy measures. We demonstrated an improved performance in detection of causative SNPs in various simulation studies. While Fuzzy MDR demonstrated higher power than that of MDR, its performance is highly dependent on the several tuning parameters. In real applications, it is not easy to choose appropriate tuning parameter values.

Here, we propose an empirical fuzzy MDR (EF-MDR), which does not require choosing optimal values of tuning parameters. EF-MDR is an empirical approach to estimating the membership degree directly from the data. In EF-MDR, the membership degree is estimated by the maximum likelihood estimator of the proportion of cases(controls) in each genotype combination. We also show that the balanced accuracy measure derived from this new membership degree estimator is a linear function of the standard chi-square test statistics. This relationship allows us to perform the standard significance test using *p*-values in the MDR framework. Details of EF-MDR are described in the Methods section with a brief review of Fuzzy MDR. A performance of the EF-MDR is assessed by comparisons with the MDR and Fuzzy MDR with two recommended parameters using two simulation categories of datasets with and without marginal effects. Finally, we analyzed Crohn’s disease (CD) and bipolar disorder (BD) data in the Wellcome Trust Case Control Consortium [42] (WTCCC) dataset using EF-MDR for detections of GGIs associated with the CD and BD.

## Methods

### Review of Fuzzy MDR

The key idea of MDR is that we can reduce dimensionality from multiple genotypes to two H/L groups by a binary classifier. As aforementioned, there are many extensions of MDR focused on the H/L binary classification. For example, if there is a genotype with the ration of cases and controls close to a threshold of the H/L classification, then this genotype can easily be misclassified. Some methods proposed the third group to overcome this drawback, but they are still limited to discrete (ternary) classification.

In order to overcome the limitation of binary classifications, we proposed Fuzzy MDR in a previous study using the fuzzy set theory. The fuzzy set theory is suggested by Zadeh [43] as an extension of the classical set. In the fuzzy set theory, an element can belong to multiple sets simultaneously by membership degrees of the multiple sets. In the Fuzzy MDR, H/L groups are fuzzy sets and samples are elements of the fuzzy set.

**Fig. 1:**
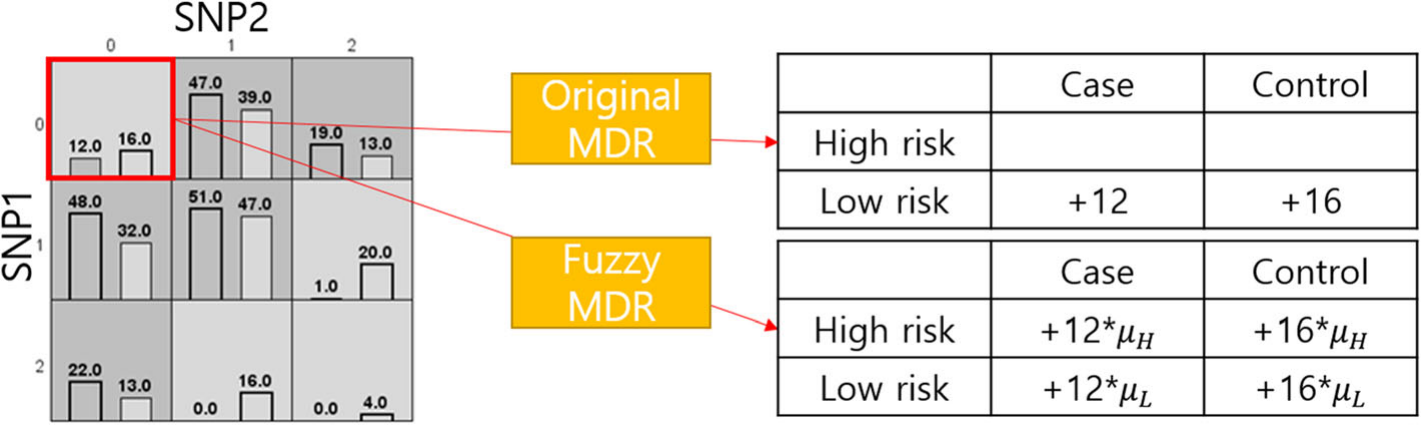
Comparison between the original MDR and the Fuzzy MDR

Let there be *n*_*i*1_ case and *n*_*i*0_ control samples who have the *i* ^*th*^ genotype. If an interaction order of a SNP combination is *k* and SNPs are biallelic, then *i* is a value among 1 ~ 3^*k*^. In the first step of Fuzzy MDR, membership degree of each genotype is calculated by *n*_*i*1_, *n*_*i*0_, *n*_*i+*1_ (total cases) and *n*_*i*+0_ (total controls) using a membership function with tuning parameters. After the membership degree calculations, 3^*k*^ genotypes are reduced to H/L groups by membership degrees *μ*_*H*_ and *μ*_*L*_ = (1-*μ*_*H*_), as shown in Fig. 1.

For illustrative purposes, consider two SNP combinations denoted as SNP1 and SNP2, as shown in Fig. 1. These two SNP combination constructs a 3 × 3 contingency table in which a cell represents a genotype combination. In each cell, there are two bars; the left dark gray bar with its value representing the number of cases, while the light gray bar with its value representing the number of control samples. For example, the first cell (0,0) contains 12 cases and 16 controls. In the original MDR method, the 12 cases and 16 controls in the first cell (0,0) completely belong to low risk (L) group. Therefore, 12 cases are stacked on the false negative (case but low risk) and 16 controls are added on the true negative (control and low risk). In the Fuzzy MDR, each sample is allowed to have partial membership of H/L groups simultaneously. Let *μ*_*H*_ and *μ*_*L*_ denote the membership degree values of the H and L, respectively. Then, *μ*_*H*_s of 12 cases are summed to the true positive count (*TP*_*Fuzzy*_) and the *μ*_*L*_s of 12 cases are summed to the false negative count (*FN*_*Fuzzy*_). In a similar manner, 16 controls are added to the false positive (*FP*_*Fuzzy*_) or the true negative (*TN*_*Fuzzy*_).

In the Fuzzy MDR, several accuracy measures were introduced such as sensitivity, specificity and balanced accuracy defined by

$$ \begin{array}{l} SE{N}_{Fuzzy}= T{P}_{Fuzzy}/\left( T{P}_{Fuzzy}+ F{N}_{Fuzzy}\right),\hfill \\ {} SP{E}_{Fuzzy}= T{N}_{Fuzzy}/\left( F{P}_{Fuzzy}+ T{N}_{Fuzzy}\right),\kern0.5em \mathrm{and}\hfill \\ {} B{A}_{Fuzzy}=\left( SE{N}_{Fuzzy}+ SP{E}_{Fuzzy}\right)/2.\hfill \end{array} $$

The best SNP combinations are selected by using one of these measures and cross validation consistencies via cross validation.

In Fuzzy MDR analysis, four optional parameters need to be specified. The first parameter is a selection of a membership function between linear and sigmoid functions. The second parameter is the use of odds ratio or standardized odds ratio. The third parameter is for a weight function. Lastly, the fourth parameter is a threshold value of membership function for defining H/L. We tested 80 parameter settings for finding the best parameter values. After the analysis of the simulation experiments, we recommended two parameter settings: the first one is a linear membership function without standardization of the odds ratio, without weight and with third threshold odd ratio value denoted by F(L,0,0,3) and the second one is a sigmoid membership function with standardization of odds ratio, with weight 1 and with the second threshold of standardized odds ratio denoted by F(S,1,1,2).

### EF-MDR

In the Fuzzy MDR, we confirmed that power is improved in the detection of causative SNPs in simulation studies. Additionally, interpretation of interaction is more flexible than MDR methods. However, choosing various tuning parameters without a golden standard is a drawback in the analysis using Fuzzy MDR. In other words, different results are produced by different parameter values.

Therefore, we propose estimating membership degrees by using the maximum likelihood estimation. Suppose there are *n*_*i*1_ case and *n*_*i*0_ control samples who have the *i* ^*th*^ genotype. A maximum likelihood estimator (MLE) for the probability of case with the *i* ^*th*^ genotype *p*_*i*1_ is *n*_*i*1_/(*n*_*i*1_ + *n*_*i*0_) under the binomial distributional assumption, and we use it as a membership degree *μ*_*H*_ of high risk (H) group. For example, if there are six cases and four controls in a cell (genotype), then a membership degree of H *μ*_*H*_ is 0.6 and a membership degree of L *μ*_*L*_ is 0.4 for the cell. There are no additional tuning parameters.

Using the MLE of the membership degree, the frequencies of true positive (TP), false negative (FN), true negative (TN) and false positive (FP) are derived as follows:

$$ \begin{array}{l} T{P}_{Fuzzy}={\displaystyle \sum }{n}_{i1}{\mu}_H(i)={\displaystyle \sum }{n}_{i1}\frac{n_{i1}}{n_{i+}}={\displaystyle \sum}\frac{{n_{i1}}^2}{n_{i+}},\hfill \\ {} F{N}_{Fuzzy}={\displaystyle \sum }{n}_{i1}{\mu}_L(i)={\displaystyle \sum }{n}_{i1}\left(1-\frac{n_{i1}}{n_{i+}}\right)={\displaystyle \sum }{n}_{i1}-{\displaystyle \sum}\frac{{n_{i1}}^2}{n_{i+}}\hfill \\ {} T{N}_{Fuzzy}={\displaystyle \sum }{n}_{i0}{\mu}_L(i)={\displaystyle \sum }{n}_{i0}\frac{n_{i0}}{n_{i+}}={\displaystyle \sum}\frac{{n_{i0}}^2}{n_{i+}},\hfill \\ {} F{P}_{Fuzzy}={\displaystyle \sum }{n}_{i0}{\mu}_H(i)={\displaystyle \sum }{n}_{i0}\left(1-\frac{n_{i0}}{n_{i+}}\right)={\displaystyle \sum }{n}_{i0}-{\displaystyle \sum}\frac{{n_{i0}}^2}{n_{i+}}.\hfill \end{array}, $$

Then, the accuracy measures such as sensitivity, specificity, and BA are defined accordingly as follows:

$$ \begin{array}{l} SE{N}_{Fuzzy}=\frac{T{P}_{Fuzzy}}{T{P}_{Fuzzy}+ F{N}_{Fuzzy}}=\frac{{\displaystyle \sum}\frac{{n_{i1}}^2}{n_{i+}}}{{\displaystyle \sum}\frac{{n_{i1}}^2}{n_{i+}}+{\displaystyle \sum }{n}_{i1}-{\displaystyle \sum}\frac{{n_{i1}}^2}{n_{i+}}}=\frac{{\displaystyle \sum}\frac{{n_{i1}}^2}{n_{i+}}}{{\displaystyle \sum }{n}_{i1}}=\frac{1}{n_{+1}}{\displaystyle \sum}\frac{{n_{i1}}^2}{n_{i+}},\hfill \\ {} SP{E}_{Fuzzy}=\frac{T{N}_{Fuzzy}}{T{N}_{Fuzzy}+ F{P}_{Fuzzy}}=\frac{{\displaystyle \sum}\frac{{n_{i0}}^2}{n_{i+}}}{{\displaystyle \sum}\frac{{n_{i0}}^2}{n_{i+}}+{\displaystyle \sum }{n}_{i0}-{\displaystyle \sum}\frac{{n_{i0}}^2}{n_{i+}}}=\frac{{\displaystyle \sum}\frac{{n_{i0}}^2}{n_{i+}}}{{\displaystyle \sum }{n}_{i0}}=\frac{1}{n_{+0}}{\displaystyle \sum}\frac{{n_{i0}}^2}{n_{i+}},\hfill \\ {} B{A}_{Fuzzy}=\frac{1}{2}\left( SE{N}_{Fuzzy}+ SP{E}_{Fuzzy}\right) = \frac{1}{2}\left(\frac{1}{n_{+1}}{\displaystyle \sum}\frac{{n_{i1}}^2}{n_{i+}}+\frac{1}{n_{+0}}{\displaystyle \sum}\frac{{n_{i0}}^2}{n_{i+}}\right).\hfill \end{array} $$

We select the best SNP combination with the highest *BA*_*Fuzzy*_ value. It can be easily shown that *BA*_*Fuzzy*_ can be expressed as a linear function of the chi-square statistics (Appendix). That is,

$$ {X}^2={n}_{++}\left(2* B{A}_{Fuzzy}-1\right). $$

In other words, we can calculate the chi-square statistics from *BA*_*Fuzzy*_ and vice versa. The degree of freedom of the chi-square statistic is the number of genotypes minus 1.

This relationship provides several advantages to EF-MDR. First, when the sample size is large, the *p*-values of *BA*_*Fuzzy*_ from EF-MDR can be calculated without permutation tests. The permutation test in MDR framework usually requires a heavy computational burden in multi-locus interactions. Second, the *p*-values can be used for the comparison of multi-locus models with different orders, providing more objective comparison results than when testing accuracy measures or cross validation consistency measure. Third, cross-validation (CV) for evaluating multi-locus models is not generally required and thus can be omitted. This omission of CV greatly reduces the execution time and removes the random variation caused by CV.

With these advantages, EF-MDR provides a more intuitive interpretation of interaction analysis than the chi-square test via visual interface of MDR. Instead of two colors used in MDR, EF-MDR represents the membership degree with different colors (figures in the Results section for representations of interaction models). More details of interpretations of EF-MDR analysis will be given in Results section.

For a SNP combination, EF-MDR first counts numbers of cases and controls in each genotype. Then, membership degrees, a *BA*_*Fuzzy*_, a chi-square value and its *p*-value are calculated sequentially. These values are calculated for all SNP combinations from two to *k*-locus, and the lowest *p*-value SNPs are selected as the best SNP combination associated with a phenotype.

## Results

First, we checked type I error rates of the EF-MDR with the null data. Second, we compared EF-MDDR with the original MDR and Fuzzy MDR in terms of power of detecting causative SNPs from two data sets with/without marginal effects. For Fuzzy MDR, two optimal sets of tuning parameter values were used. Finally, we applied our EF-MDR to WTCCC data to detect interactions associated with Crohn’s disease and bipolar disorder.

### Type I error

To check for type I error, we used a simulation dataset in [21] and used non-causative SNPs. This dataset consists of four sample sizes: 200, 400, 800 and 1600. For each sample size, there are 100 replicates for 70 different genetic models. For each dataset there are two causative SNPs and 998 non-causative SNPs. For a given sample size, we randomly selected two SNPs among non-causative SNPs from each dataset and calculated 7000 *p*-values. The type I error rate was calculated as a proportion of datasets with *p*-values smaller than the threshold value.

In Table 1, the type I error rates are lower than the threshold values when the sample size is 200. However, the differences between type I error rates and threshold values tend to reduce as the sample size increases. Type I error rates of 1600 samples are very close to the threshold values. This phenomenon is caused by the fact that the chi-square test approximates the chi-square distribution better for larger sample sizes.

**Table 1:** Type I error rate of EF-MDR

Threshold	Number of samples			
	200	400	800	1600
0.010	0.004	0.006	0.009	0.008
0.050	0.032	0.039	0.044	0.050
0.100	0.072	0.090	0.093	0.102

### Simulation experiment without marginal effects

**Fig. 2:**
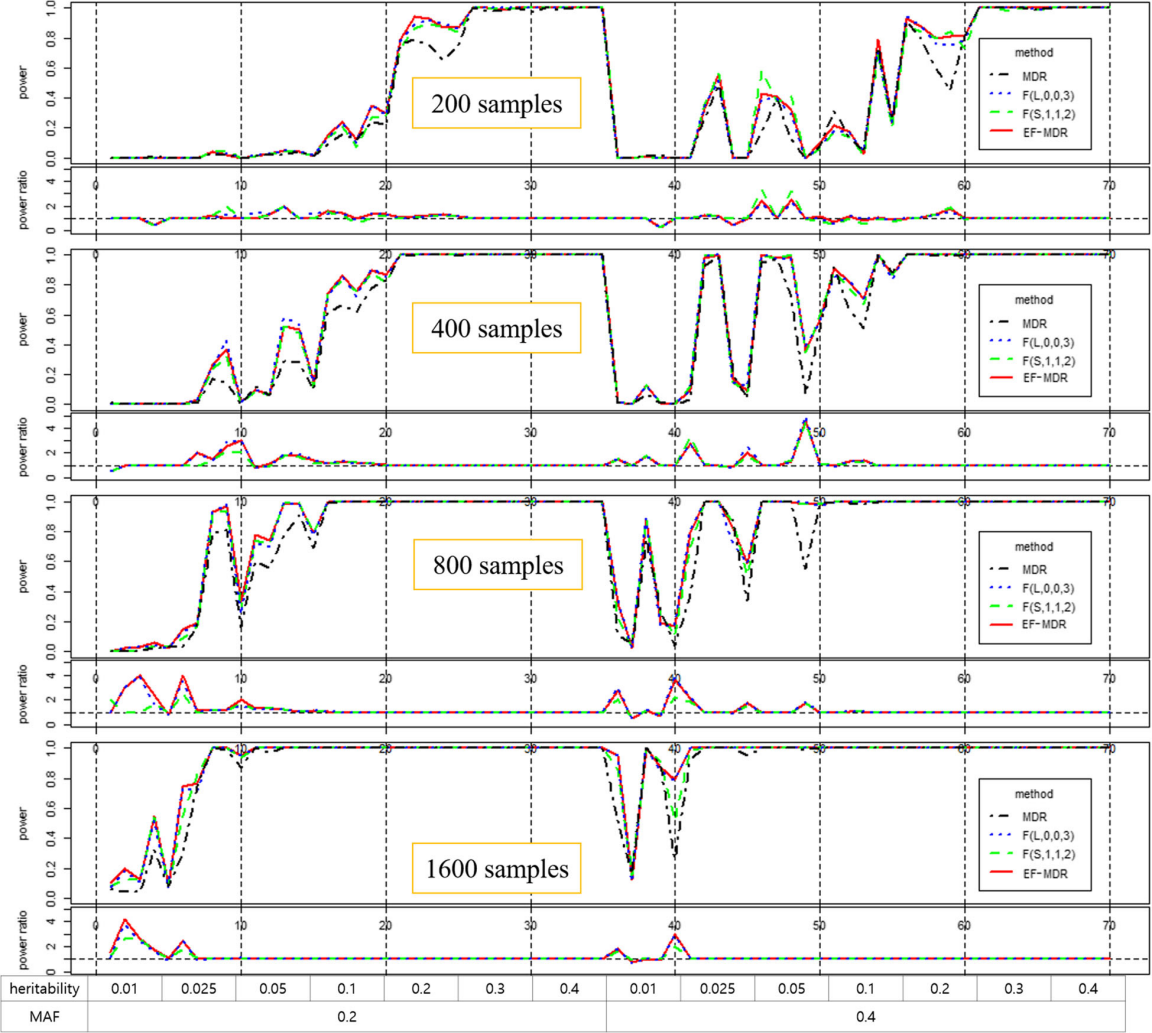
Power comparison of experiments without marginal effects

We used the simulation data without marginal effects [21]. The dataset consists of four sample sizes and genotype information of 1000 SNPs. Among 1000 SNPs, two SNPs are causative SNPs, and the other SNPs are non-causative SNPs. The two causative SNPs were generated based on 70 penetrance tables, and each penetrance table is calculated with a combination of seven heritability values, two minor allele frequency (MAF) and five interaction models. For each penetrance table, 100 data are generated. The results are summarized in Fig. 2.

In Fig. 2, power is defined as the ratio of successful finding of the pre-defined causative two SNPs in one hundred data, and power ratio is the ratio of power of each method to power of MDR. Powers of MDR are lower than other methods in most combinations of sample sizes, heritability values and MAFs. As illustrated in Fig. 2, the powers of Fuzzy MDRs show frequent fluctuation. While it was hard to decide which one performs the best, EF-MDR showed higher average powers than two Fuzzy MDR methods for each sample size. Additionally, the average power of EF-MDR was shown to be higher than those of Fuzzy MDRs. Although it is not guaranteed that EF-MDR always yields higher power than Fuzzy MDR, EF-MDR has the advantage of providing more stable and robust results to tuning parameters.

### Simulation experiment with marginal effects

**Fig. 3:**
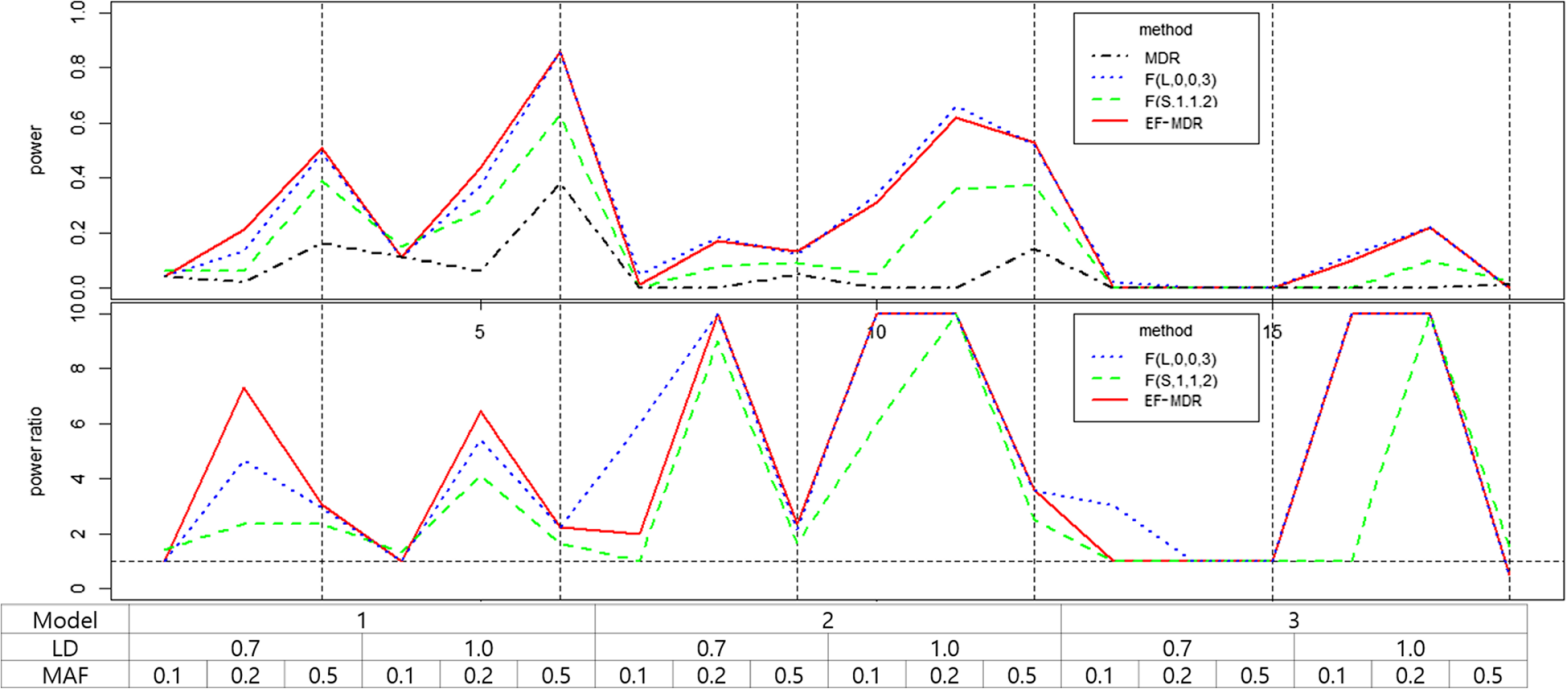
Power comparison of experiments with marginal effects

We used the datasets with marginal effects generated from previous studies [44–46]. The datasets consist of three interaction models, three MAF values and linkage disequilibrium (LD) values. Totally, 18 datasets were generated using the same methods with same parameter values. Model 1 is a ‘multiplicative effect between and within loci’ model (additive model) and it assumes that the relative risk is exponentially increased by the total number of minor alleles of two SNPs. Model 2 is a ‘multiplicative effect between loci’ model (multiplicative model) and it assumes that the relative risk is exponentially increased by the product of number of minor alleles of each SNP. Model 3 is a threshold model and it assumes that the relative risk is consistently increased in genotype combinations which have at least one minor allele of both SNPs. These three models are widely used to evaluate performance of GGI methods [44–46]. Each dataset consists of one hundred replicates. For the simplicity, we fixed sample size 4000 with 2000 cases and 2000 controls. The results are summarized in Fig. 3.

Figure 3 shows the power improvements of two Fuzzy MDRs and EF-MDR over MDR in most models, LDs and MAFs. Among Fuzzy MDRs, F(S,1,1,2) is relatively lower than the others and powers of F(L,0,0,3) and EF-MDR look similar. The average power (0.2389) of EF-MDR is slightly higher than average power (0.2350) of F(L,0,0,3).

### Real data experiment

We applied the EF-MDR to a Crohn’s disease (CD) and a bipolar disorder in Wellcome Trust Case Control Consortium (WTCCC) data [42]. The CD data in WTCCC data consists of about 500,000 genotype information of 1949 cases and 3004 controls. For an illustrative purpose, we selected 30 SNPs reported to have association with CD in previous studies [42, 47, 48]. We summarized basic characteristics of each SNP in Table 2. The p-values of Table 2 are calculated by chi-square tests of association between individual SNP and CD status.

**Table 2:** Basic characteristics of each SNP for Crohn’s disease (CD)

Index	rs number	MAF	Chromosome (gene)	*p*-value (rank)	Index	rs number	MAF	Chromosome (gene)	*p*-value (rank)
1	rs11805303	0.347	1 (IL23R)	4.41E-13 (2)	16	rs1456893	0.304	7	4.02E-05 (19)
2	rs12035082	0.410	1	2.70E-07 (8)	17	rs4263839	0.313	9 (NFSF15)	1.64E-05 (17)
3	rs10801047	0.079	1	1.09E-05 (15)	18	rs17582416	0.363	10 (OC105376492)	1.11E-03 (23)
4	rs11584383	0.297	1 (MROH3P)	4.62E-05 (20)	19	rs10995271	0.413	10	1.54E-05 (16)
5	rs3828309	0.453	2 (ATG16L1)	1.29E-13 (1)	20	rs10883365	0.498	10 (INC01475)	1.60E-06 (11)
6	rs9858542	0.299	3 (BSN)	3.20E-07 (9)	21	rs7927894	0.408	11	1.28E-02 (28)
7	rs17234657	0.146	5	1.71E-12 (3)	22	rs11175593	0.017	12 (OC105369735)	4.22E-02 (30)
8	rs9292777	0.367	5	1.04E-11 (4)	23	rs3764147	0.222	13 (LACC1)	3.34E-06 (13)
9	rs10077785	0.220	5 (C5orf56)	6.39E-05 (22)	24	rs17221417	0.310	16 (NOD2)	2.81E-10 (5)
10	rs13361189	0.084	5	7.04E-08 (6)	25	rs2872507	0.491	17	1.24E-03 (24)
11	rs4958847	0.130	5 (IRGM)	1.81E-06 (12)	26	rs744166	0.422	17 (STAT3)	6.27E-05 (21)
12	rs11747270	0.099	5 (IRGM)	3.13E-05 (18)	27	rs2542151	0.181	18	1.74E-07 (7)
13	rs6887695	0.329	5	4.69E-03 (27)	28	rs1736135	0.412	21 (LOC101927745)	3.39E-02 (29)
14	rs6908425	0.214	6 (CDKAL1)	1.02E-06 (10)	29	rs2836754	0.374	21 (LOC400867)	5.67E-06 (14)
15	rs7746082	0.293	6	4.20E-03 (26)	30	rs762421	0.408	21 (LOC105377139)	2.35E-03 (25)

We performed EF-MDR analyses from single-locus to five-locus and summarized results in Table 3. In spite of ultimately low *p*-values, the values of *BA*_*FUZZY*_ are approximately 0.5. Most SNP combinations include SNP5. Note that SNP5 showed the most significant result in single SNP analysis (order = 1). SNP5 disappeared in the result of two-locus (order = 2) and reappeared in the results of higher-orders (order = 3, 4, and 5). In addition, we applied MDR to the CD data for comparison purpose with EF-MDR. The MDR results are summarized in terms of balanced accuracy (BA), sensitivity (SEN) and specificity (SPE). We did not compute the *p*-values for MDR, because it takes too much time to compute the *p*-values by permutation with a high precision of 1.0E-10. As shown in Table 3, most prediction measures of EF-MDR have smaller values than those of MDRs. However, these measures are not directly comparable, because their distributions differ much. Instead, the use of *p*-values is more appropriate to choose the appropriate SNP combinations. Note that the p-values of EF-MDR can be easily computed by using the linear relationship between *BA*_*FUZZY*_s and the chi-square statistics.

**Table 3:** Results of Crohn’s disease (CD) data analysis

order	SNP combination	EF-MDR				MDR		
		*BA*_*FUZZY*_	*p*-value	*SEN*_*FUZZY*_	*SPE*_*FUZZY*_	BA	SEN	SPE
1	5	0.5060	1.292E-13	0.4002	0.6121	0.5494	0.3563	0.7425
2	1, 8	0.5121	6.211E-22	0.4069	0.6171	0.5664	0.5625	0.5702
3	1, 5, 8	0.5184	4.715E-25	0.4141	0.6224	0.5807	0.5203	0.6411
4	1, 5, 8, 23	0.5290	2.251E-24	0.4263	0.6319	0.5987	0.5557	0.6417
5	5, 8, 18, 24, 29	0.5518	2.480E-18	0.4585	0.6452	0.6219	0.5625	0.6814

**Fig. 4:**
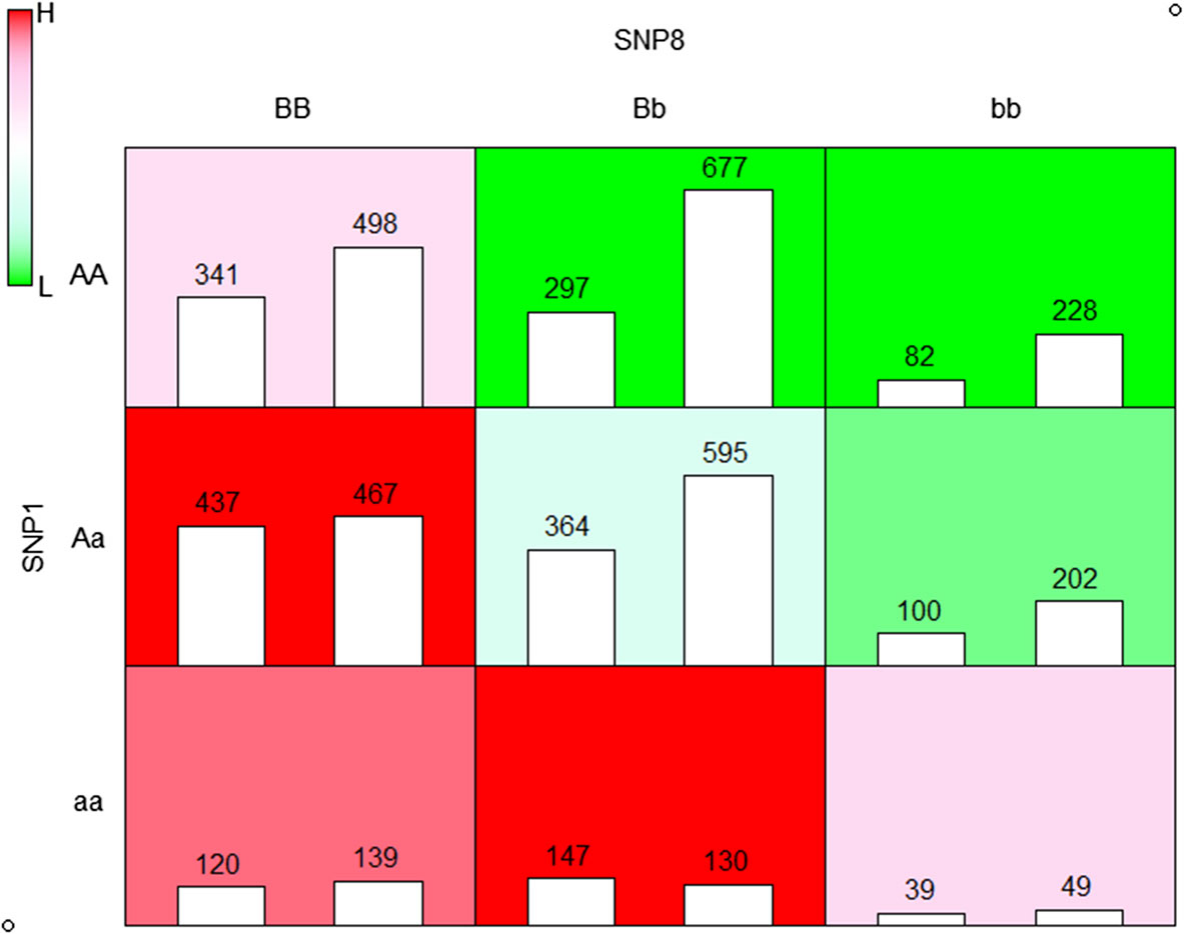
Representation of the interaction between SNP1 and SNP8 for CD

**Fig. 5:**
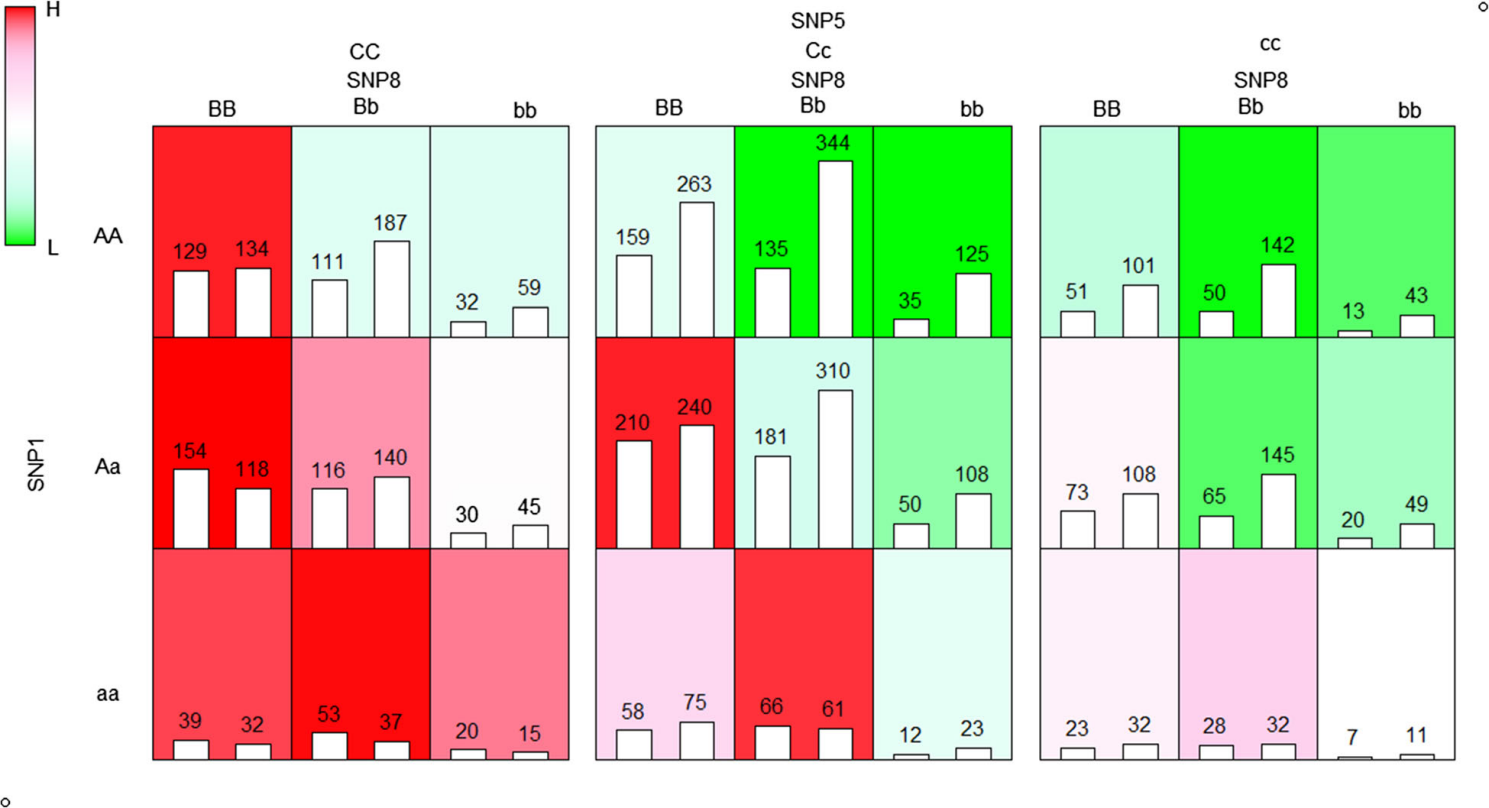
Representation of the interaction among SNP1, SNP5 and SNP8 for CD

Among the results in Table 3, we selected the two-locus and three-locus SNP combinations and represented them in Figs. 4 and 5, respectively. The three-locus SNP combination model is the most significant, but it is hard to derive their biological interpretation from interaction patterns. Therefore, we analyzed a less complex interaction of the two-locus SNP combination at first.

In Figs. 4 and 5, the uppercase alphabets represent major allele and lowercase alphabets represent minor allele. That is, ‘A’ or ‘a’ represent major and minor allele of the first SNP respectively, and ‘B’ or ‘b’ represent allele of the second SNP, and so on. In each cell, there are two bars; the left bar with its value represents the number of cases, while the right bar with its value represents the number of control samples. Background colors represent the degree of membership function. Red background color means high-risk group and the green background color low-risk group. The darker the color, the larger the membership value is; the lighter the color, the smaller the membership value. The white background color means that the membership degrees of H and L are similar.

Figure 4 represents the interaction result of two-locus SNP combination of SNP1 and SNP8. There are some interesting interpretations available. First, four green colored cells (SNP1,SNP8) = (AA,Bb), (AA,bb), (Aa,Bb) and (Aa,bb) are considered to belong to the low-risk (L) group and the other cells to the high-risk (H) group. Note that this interaction model corresponds to M27 in two-locus disease models [17], called ‘jointly dominant-dominant model (DD)’ and is considered as one of important interaction models in earlier studies [49–51]. Second, three dark red cells (SNP1,SNP8) = (Aa,BB), (aa,BB) and (aa,Bb) are considered to belong to H with strong certainty. The three diagonal cells (SNP1,SNP8) = (AA,BB), (Aa,Bb) and (aa,bb) show weak evidences of belonging to H or L. Ignoring these cells yields a new interaction model corresponding to M11 in two-locus disease models [17], called the ‘threshold model (T)’, and is also considered as one of the most important interaction models [49, 51]. Of course, possible interpretations are not limited to these binary classifications. For example, three dark cells (SNP1,SNP8) = (Aa,BB), (aa,BB) and (aa,Bb) are considered as H, three dark green cells (SNP1,SNP8) = (AA,Bb), (AA,bb) and (Aa,bb) are considered as L and three diagonal cells (SNP1,SNP8) = (AA,BB), (Aa,Bb) and (aa,bb) are considered as ‘no evidence’ or ‘unknown risk’ group.

Figure 5 represents the interaction of the three-locus SNP combination (SNP1, SNP5, SNP8). Comparison of Fig. 5 with Fig. 4 provides a more detailed interpretation of three-order interactions. Each cell in Fig. 4 showing the interaction pattern between SNP1 and SNP8 is divided into the three cells in Fig. 5. For example, the red colored cell (SNP1, SNP8) = (Aa,BB) in Fig. 4 are split into the three red colored cells (SNP1,SNP5,SNP8) = (Aa,CC,BB), (Aa,Cc,BB), (Aa,cc,BB) in Fig. 5; the green colored cell (SNP1, SNP8) = (AA,Bb) in Fig. 4 are split into the three green colored cells (SNP1,SNP5,SNP8) = (AA,CC,Bb), (AA,Cc,Bb), (AA,cc,Bb) in Fig. 5. However, the light red colored cell (SNP1, SNP8) = (AA,BB) and the light green colored cell (Aa,Bb) in Fig. 4 are split into the three cells with different colors in Fig. 5, suggesting strong three-order interactions.

In addition, Fig. 5 itself provides some interesting patterns. Figure 5 shows three two-way contingency tables of (SNP1, SNP8) for a given genotype of SNP5. From the left to right, the red colored cells disappeared, while more green colored cells appeared. In particular, three cells (SNP1,SNP5,SNP8) = (Aa,**,BB), (aa,**,BB) and (aa,**,Bb) show shades of red in a consistent manner and the colors become lighter from the left to the right, as the genotype of SNP5 changes.

In summary, Fig. 5 shows evidence of strong three-way interactions among the three SNPs. Thus, the genotypes of SNP1, SNP5 and SNP8 need to be considered simultaneously for the association analysis on the CD.

**Fig. 6:**
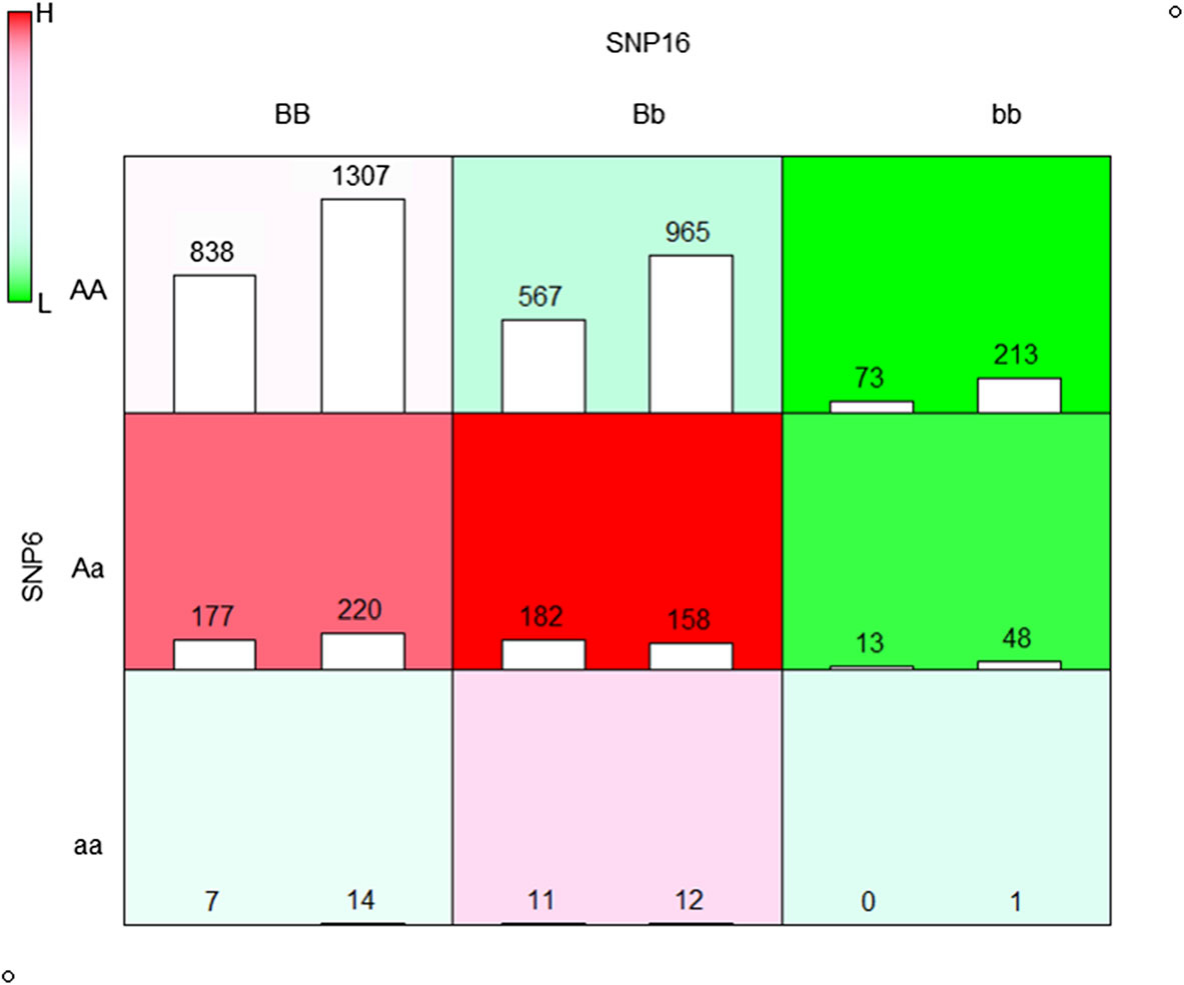
Representation of the interaction between SNP6 and SNP16 for BD

In addition, we applied EF-MDR to a bipolar disorder (BD) dataset in WTCCC. This dataset consists of about 500,000 SNPs from 1868 cases and 2938 controls. Among these SNPs, we selected 19 candidate SNPs using the same selection strategy in Jung et al. [41]. The results of the bipolar data analysis are summarized in Table 4. Aforementioned, these prediction measures are not directly comparable between EF-MDR and MDR, because their distributions differ much. In Table 4, all models of orders 2 and higher provided similar significant results. For simple interpretation, we provide the graphical representation of the interaction of two-locus SNP combination in Fig. 6.

**Table 4:** Results of the bipolar disorder (BD) data analysis

order	SNP combination	EF-MDR				MDR		
		*BA*_*FUZZY*_	*p*-value	*SEN*_*FUZZY*_	*SPE*_*FUZZY*_	BA	SEN	SPE
1	16	0.5033	1.33e-07	0.3929	0.6140	0.5216	0.9540	0.0892
2	6, 16	0.5072	6.16e-12	0.3978	0.6171	0.5345	0.6467	0.4224
3	6, 15, 16	0.5118	3.21e-13	0.4031	0.6205	0.5568	0.6146	0.4990
4	5, 15, 17, 19	0.5203	4.87e-13	0.4133	0.6270	0.5850	0.6761	0.4939
5	5, 10, 15, 17, 19	0.5376	1.06e-13	0.4347	0.6406	0.6101	0.6376	0.5827

Figure 6 represents the interaction of two-locus SNP combination SNP6 and SNP16. There is a possible interpretation of the interaction. Three dark green colored cells (SNP6,SNP16) = (AA,Bb), (AA,bb) and (Aa,bb) are considered to belong to the low-risk (L) and the other cells to the high-risk (H) group. Note that this interaction model corresponds to M95 (equivalent to M11) in two-locus disease models [17], called ‘threshold model (T)’ same as the second interpretation of interaction for two-locus SNP combination in CD data results. As aforementioned, this M11 interaction model is considered as one of the important interaction models [49, 51].

## Discussion

The MDR method consists of loading an input file and running it on a main algorithm (selection of SNP combinations, calculation of case-control ratios of each multi-locus genotype, and identification of multi-locus genotypes) on the cross-validation (CV) structure. The execution time of MDR method is exponentially increased by the number of SNP and the interaction order. Suppose there are *n*_++_ samples and *s* SNPs. The time complexity of loading an input file is *O*(*s* × *n*_*++*_) and the time complexity of a main algorithm on the *m*-fold cross-validation structure is $$ O\left( m\times \left(\begin{array}{c}\hfill s\hfill \\ {}\hfill k\hfill \end{array}\right)\times {n}_{++}\right)= O\left( m\times {s}^k\times {n}_{++}\right) $$, for the detection procedure of *k*-locus interactions. Therefore, the time complexity of the total procedure of MDR is *O*(*m* × *s* ^*k*^ × *n*_+ +_) and the omission of CV reduces the execution time to about $\frac{1}{m}$. In addition, the execution time of MDR can be increased by permutation test. On the other hand, since there is no additional computation burden in our EF-MDR method, its time complexity is *O*(*s* ^*k*^ × *n*_+ +_). We performed the comparison study on computational times between MDR with 10-fold cross validation and EF-MDR by using a real dataset of CD with 30 SNPs of 4953 samples. The comparison result on computational times is summarized in the Table 5, which demonstrates the great computational reduction of EF-MDR over MDR. This comparison was performed using R scripts on a 64-bit MS window platform with 3.4 GHz CPU and 8 GB RAM.

**Table 5:** Execution times of MDR and EF-MDR in seconds

order	MDR	EF-MDR
1	2.99	0.29
2	61.20	3.99
3	1.01E + 03	39.79
4	1.62E + 04	3.09E + 02
5	2.78 E + 05	2.03E + 03

## Conclusion

We propose an empirical extension of Fuzzy MDR for detections and interpretations of GGIs. The proposed EF-MDR uses the proportion of cases as a membership degree. EF-MDR avoids choosing optimal tuning parameter values in real data application, while maintaining the high performance of optimal Fuzzy MDR. Through simulation studies, EF-MDR was shown to have higher power than that of Fuzzy MDR and MDR in various simulation models. In real data application, EF-MDR demonstrated its ability of providing a more flexible interpretation of biologically meaningful interactions.

We also showed a linear relationship between the balanced accuracy measure of EF-MDR and the standard chi-square statistics. This relationship provides a great advantage of reducing a computational burden. The *p*-values can be easily computed from the chi-square distribution, which enables EF-MDR to avoid not only cross-validation for selecting the best SNP combinations, but also permutation for calculating *p*-values.

Furthermore, EF-MDR inherits all the merits of MDR and Fuzzy MDR. All kinds of GGI interpretation made by MDR can also be made in EF-MDR. In addition, each cell derived from the genotype combination has its own membership degrees, which enables researchers to detect more biologically plausible GGI, as Fuzzy MDR does. EF-MDR can be easily incorporated into the existing MDR extensions such as generalized MDR (GMDR) [22] and quantitative MDR (QMDR) [52].

## Appendix

A chi-square statistic of association test between genotypes and a phenotype is ${\displaystyle \sum}\frac{{\left({\mathrm{n}}_{\mathrm{i}0} - {\mathrm{e}}_{\mathrm{i}0}\right)}^2}{{\mathrm{e}}_{\mathrm{i}0}}+{\displaystyle \sum}\frac{{\left({\mathrm{n}}_{\mathrm{i}1} - {\mathrm{e}}_{\mathrm{i}1}\right)}^2}{{\mathrm{e}}_{\mathrm{i}1}}.$

For cases, ${\mathrm{e}}_{\mathrm{i}1}={\mathrm{n}}_{\mathrm{i}+}\frac{{\mathrm{n}}_{+1}}{\mathrm{n}++}$. Then,

$$ \begin{array}{l}{\displaystyle \sum}\frac{{\left({\mathrm{n}}_{\mathrm{i}1} - {\mathrm{e}}_{\mathrm{i}1}\right)}^2}{{\mathrm{e}}_{\mathrm{i}1}}={\displaystyle \sum}\frac{{\left({\mathrm{n}}_{\mathrm{i}1} - {\mathrm{n}}_{\mathrm{i}+}\frac{{\mathrm{n}}_{+1}}{\mathrm{n}++}\right)}^2}{{\mathrm{n}}_{\mathrm{i}+}\frac{{\mathrm{n}}_{+1}}{\mathrm{n}++}}={\displaystyle \sum}\frac{{{\mathrm{n}}_{\mathrm{i}1}}^2-2{\mathrm{n}}_{\mathrm{i}1}{\mathrm{n}}_{\mathrm{i}+}\frac{{\mathrm{n}}_{+1}}{\mathrm{n}++}+{\left({\mathrm{n}}_{\mathrm{i}+}\frac{{\mathrm{n}}_{+1}}{\mathrm{n}++}\right)}^2}{{\mathrm{n}}_{\mathrm{i}+}\frac{{\mathrm{n}}_{+1}}{\mathrm{n}++}}={\displaystyle \sum}\frac{{{\mathrm{n}}_{\mathrm{i}1}}^2}{{\mathrm{n}}_{\mathrm{i}+}\frac{{\mathrm{n}}_{+1}}{\mathrm{n}++}}-\\ {}{\displaystyle \sum}\frac{2{\mathrm{n}}_{\mathrm{i}1}{\mathrm{n}}_{\mathrm{i}+}\frac{{\mathrm{n}}_{+1}}{\mathrm{n}++}}{{\mathrm{n}}_{\mathrm{i}+}\frac{{\mathrm{n}}_{+1}}{\mathrm{n}++}}+{\displaystyle \sum}\frac{{\left({\mathrm{n}}_{\mathrm{i}+}\frac{{\mathrm{n}}_{+1}}{\mathrm{n}++}\right)}^2}{{\mathrm{n}}_{\mathrm{i}+}\frac{{\mathrm{n}}_{+1}}{\mathrm{n}++}}\\ {}=\frac{{\mathrm{n}}_{++}}{{\mathrm{n}}_{+1}}{\displaystyle \sum}\frac{{{\mathrm{n}}_{\mathrm{i}1}}^2}{{\mathrm{n}}_{\mathrm{i}+}}-{\displaystyle \sum }2{\mathrm{n}}_{\mathrm{i}1}+{\displaystyle \sum }{\mathrm{n}}_{\mathrm{i}+}\frac{{\mathrm{n}}_{+1}}{\mathrm{n}++}=\frac{{\mathrm{n}}_{++}}{{\mathrm{n}}_{+1}}{\displaystyle \sum}\frac{{{\mathrm{n}}_{\mathrm{i}1}}^2}{{\mathrm{n}}_{\mathrm{i}+}}-2{\mathrm{n}}_{+1}+{\mathrm{n}}_{+1}=\frac{{\mathrm{n}}_{++}}{{\mathrm{n}}_{+1}}{\displaystyle \sum}\frac{{{\mathrm{n}}_{\mathrm{i}1}}^2}{{\mathrm{n}}_{\mathrm{i}+}}-{\mathrm{n}}_{+1}.\end{array} $$

Similar manner, ${\displaystyle \sum}\frac{{\left({\mathrm{n}}_{\mathrm{i}0} - {\mathrm{e}}_{\mathrm{i}0}\right)}^2}{{\mathrm{e}}_{\mathrm{i}0}}=\frac{{\mathrm{n}}_{++}}{{\mathrm{n}}_{+0}}{\displaystyle \sum}\frac{{{\mathrm{n}}_{\mathrm{i}0}}^2}{{\mathrm{n}}_{\mathrm{i}+}}-{\mathrm{n}}_{+0}$. Then, the chi-square statistic value is

$$ \frac{{\mathrm{n}}_{++}}{{\mathrm{n}}_{+1}}{\displaystyle \sum}\frac{{{\mathrm{n}}_{\mathrm{i}1}}^2}{{\mathrm{n}}_{\mathrm{i}+}}-{\mathrm{n}}_{+1}+\frac{{\mathrm{n}}_{++}}{{\mathrm{n}}_{+0}}{\displaystyle \sum}\frac{{{\mathrm{n}}_{\mathrm{i}0}}^2}{{\mathrm{n}}_{\mathrm{i}+}}-{\mathrm{n}}_{+0}{\mathrm{n}}_{++}\left(\frac{1}{{\mathrm{n}}_{+1}}{\displaystyle \sum}\frac{{{\mathrm{n}}_{\mathrm{i}1}}^2}{{\mathrm{n}}_{\mathrm{i}+}}+\frac{1}{{\mathrm{n}}_{+0}}{\displaystyle \sum}\frac{{{\mathrm{n}}_{\mathrm{i}0}}^2}{{\mathrm{n}}_{\mathrm{i}+}}\right)-{\mathrm{n}}_{++}={\mathrm{n}}_{++}\left(2*{\mathrm{BA}}_{\mathrm{Fuzzy}}-1\right). $$
